# Marijuana Consumption and Reactivity Are Positively Associated with the Fading Affect Bias for Marijuana Events in Person and Online

**DOI:** 10.3390/bs16040611

**Published:** 2026-04-20

**Authors:** Jeffrey Alan Gibbons, Chayse Angela Cotton, Matthew Traversa, Emma Friedmann, Kaylee Harris

**Affiliations:** 1 Department of Psychology, Christopher Newport University, 1 Avenue of the Arts, Newport News, VA 23606, USA; chayse.cotton.24@cnu.edu (C.A.C.); matthew.traversa.19@cnu.edu (M.T.); kaylee.harris.18@cnu.edu (K.H.); 2 Department of Psychology, Florida State University, 288 Champions Way, Tallahassee, FL 32306, USA; friedmann@psy.fsu.edu

**Keywords:** fading affect bias, marijuana consumption, marijuana events, healthy coping

## Abstract

The fading affect bias (FAB) is the faster fading of unpleasant than pleasant affect, and this effect is positively and negatively related to healthy/adaptive and unhealthy/non-adaptive outcomes, respectively. Research has argued that the FAB makes people happy and it prompts them to seek out pleasant experiences. Although Pillersdorf and Scoboria found a negative relation between the FAB and marijuana consumption, they only examined non-marijuana events. This investigation was limited because the relation between the FAB and marijuana consumption may be absent or positive for marijuana events. The current study examined the relation of the FAB to marijuana consumption measures across marijuana and non-marijuana events. The study was conducted both in person (Experiment 1; *n* = 328) and online (Experiment 2; *n* = 232). Analyses included ANOVAs to examine fading affect across initial event affect and event type, and Process Model 1 was used to evaluate the fading affect bias across initial event affect in 2-way interactions with continuous variables. Process Model 3 was used to investigate fading affect across initial event affect and event type in 3-way interactions with continuous variables. Both experiments showed a robust FAB that was positively related to adaptive variables and negatively related to non-adaptive variables, and it was positively related to marijuana consumption/reactivity. In addition, the positive relations between FAB and marijuana consumption (hours) and reactivity (highness) measures in Experiment 1 and a marijuana reactivity measure (highness) in Experiment 2 were only found for marijuana events. Implications, limitations, and future research directions are discussed.

## 1. Introduction

While cigarette smoking has been studied by the medical profession for its cancer-causing effects (e.g., Inoue-Choi et al., 2018), the emotional costs of marijuana consumption have been presented in the psychological literature. For example, Degenhardt et al. (2003) found that heavy marijuana users reported more severe levels of depression compared to less frequent users, and Dorard et al. (2008) showed that cannabis abusers reported higher levels of trait anxiety than non-users. More recently, Pillersdorf and Scoboria (2019) found that marijuana consumption was negatively related to emotional regulation in the form of the fading affect bias (FAB), which is the faster fading of unpleasant than pleasant affect related to events in autobiographical memory (Walker et al., 1997). One reason that marijuana consumption may have resulted in a low FAB in the Pillersdorf and Scoboria study is that participants were only asked to remember and rate non-marijuana events. The relation between the FAB and marijuana consumption could be absent or positive for marijuana events, especially considering recent evidence that marijuana consumption positively predicts adaptive outcome measures (Castle et al., 2025; Sznitman et al., 2022). To correct this oversight, the goal of the current study was to examine the FAB and the relation of the FAB to marijuana consumption/reactivity across marijuana and non-marijuana events.

### 1.1. The Fading Affect Bias

The fading affect bias (FAB; Walker et al., 2003) is the tendency for negative affect to fade faster than positive affect from autobiographical memory. Though the FAB received its name in 2003, it has been studied for nearly a century (Cason, 1932; Holmes, 1970; Jersild, 1931; Meltzer, 1930, 1931; Walker et al., 1997; Watters & Leeper, 1936). The FAB begins within 12 h of events, remains constant for 3 months (Gibbons et al., 2011), and increases from that point (Walker et al., 1997). The FAB is also robust across various cultures (Ritchie et al., 2015), makes people feel good, and helps them seek new experiences (Ritchie et al., 2015; Walker et al., 2003; Walker & Skowronski, 2009).

Theories relevant for the FAB suggest that biological, cognitive, and emotional resources reduce the harmful effects of unpleasant events (Taylor, 1991) by putting them in perspective, which minimizes their impact (Ritchie et al., 2015). Conversely, these bio-social-psychological resources seem to enhance positive self-perceptions with a focus on the importance of pleasant events (Ritchie et al., 2015; Sedikides & Alicke, 2018). The FAB helps people feel good about themselves (e.g., Ritchie et al., 2015), which is congruent with self-enhancement theories (Ritchie et al., 2006; Sedikides & Alicke, 2018). Together, these assertions suggest that the FAB is a general healthy coping outcome. Similar research demonstrated that event rehearsal ratings positively predicted the FAB (e.g., Skowronski et al., 2004). In contrast to the smaller body of research demonstrating positive relations between the FAB and healthy outcomes, a larger body of research shows negative relations between the FAB and unhealthy outcomes. Specifically, the FAB is negatively related to dispositional mood (Ritchie et al., 2009), dysphoria (Walker et al., 2003), trait anxiety (Walker et al., 2014), state anxiety (Gibbons & Lee, 2019), stress (Gibbons & Lee, 2019), eating disorder symptoms (Ritchie et al., 2019), and parental abuse (Skowronski et al., 2016, 2023). Importantly, the FAB was smaller for marijuana consumers than marijuana non-users (Pillersdorf & Scoboria, 2019), which suggested that marijuana consumption is unhealthy.

### 1.2. Marijuana Literature Shows Bifurcated Findings

The literature surrounding marijuana contains many contradicting findings pertaining to the adaptivity of marijuana consumption. For instance, in a 4-week diary study, Gruber et al. (2012) found that marijuana use lowered daily mood ratings across time in healthy participants. Similarly, Lex et al. (1989) asked a sample of 30 participants to complete daily diary questionnaires that monitored their use of marijuana and other drugs and found that heavy marijuana users reported higher negative moods (e.g., confusion, fatigue, and anger) and lower positive moods (e.g., friendliness, elation, and vigor) than light marijuana users. In a cross-sectional study, Hines et al. (2020) found that depression, generalized anxiety disorder, and psychotic experiences were positively related to high-potency marijuana consumption in a sample of 1087 participants. Petrucci (2020) examined a sample of 1168 men and women and reported that cannabis use was positively related to apathy. Similarly, Looby and Earleywine (2007) found that a large sample (*n* = 2500) of cannabis users meeting DSM-IV-TR criteria for dependence reported low levels of motivation, and Lac and Luk (2017) reported that consumption was associated with low initiative and persistence that worsened over time in a sample of 505 college students. While those studies found marijuana consumption to be positively associated with unhealthy outcomes, providing evidence of marijuana consumption being a maladaptive outcome, other studies have found marijuana to be an adaptive outcome. For example, Roitman et al. (2014) found that the main psychoactive element in marijuana, THC, may be effective in reducing nightmares and improving sleep quality in patients with PTSD. Sznitman et al. (2022) conducted a 14-day diary study and found that intended cannabis use and recent cannabis use were related to enhanced positive affect and decreased negative affect. In addition, participants with bipolar disorder, PTSD, and depression who consumed cannabis reported improved moods. Gruber et al. (2012) found similar results in a four-week diary study where bipolar participants who consumed marijuana reported improved mood symptoms when compared to the bipolar participants who did not consume marijuana. Lake et al. (2019) uncovered similar effects in the context of PTSD in a sample of 225,113 participants where PTSD was associated with the likelihood of experiencing a major depressive episode and suicidal ideation in non-cannabis users, but the relation was not found in cannabis users. Similarly, Li et al. (2020) examined the effectiveness of cannabis to immediately relieve symptoms of depression in a sample of 1819 participants who self-administered cannabis.

The existing literature suggests that marijuana consumption is associated with both adaptive and maladaptive outcomes, with many studies (e.g., Gruber et al., 2012) finding both adaptive and maladaptive effects of marijuana consumption in clinical and healthy populations, respectively. Nevertheless, several limitations within the cited research should be noted. Much of the current research relies heavily on self-reported measures of marijuana use, which may produce bias in recall as well as other measurement areas. In addition, many of the studies employed cross-sectional designs, which limit the ability to determine the exact causal relation between marijuana use and psychological/health outcomes. Differences in dosage, potency, frequency of use and the context of consumption (medical versus recreational, flower versus vaping) may also contribute to the mixed findings observed across studies. Taken together, these considerations suggest that the effects of marijuana are complex and may depend on contextual factors and individual differences.

### 1.3. The Current Study

The FAB has been studied in many contexts, including marijuana, with Pillersdorf and Scoboria (2019) finding a larger FAB for non-marijuana consumers than marijuana consumers. Based on Taylor’s (1991) mobilization-minimization hypothesis, the consumption of and reactivity to marijuana by participants in this study likely inhibited the biological (e.g., Drazdowski et al., 2019), cognitive (e.g., Guy et al., 2004; Schuster et al., 2019), and emotional (e.g., Gruber et al., 2012; Lex et al., 1989) resources, as demonstrated in the marijuana literature, that typically minimize the sting of unpleasant experiences (Ritchie et al., 2015) and enhance self-perceptions by emphasizing pleasant incidents (Ritchie et al., 2015). Consequently, marijuana consumption muted the FAB.

The problem with the Pillersdorf and Scoboria (2019) study is that it did not examine marijuana events, which may have shown a positive relation between the FAB and marijuana consumption like that in more recent research (Castle et al., 2025; Sznitman et al., 2022). Although many FAB studies fail to instructionally manipulate event type, the ones that do often find that event type influences the FAB and its relations to other variables. For example, Skowronski et al. (2016, 2023) found similar FAB effects across participants likely and unlikely to abuse children for general events, but small FAB effects for participants likely to abuse children for events involving their children. Similarly, Muir and Brown (2024) discovered that the FAB was smaller for events related to the pandemic than events not related to the pandemic.

The current study was designed to correct the limitation in the Pillersdorf and Scoboria (2019) study by examining the FAB and its relations to adaptive and non-adaptive variables, including marijuana consumption/reactivity, across marijuana and non-marijuana events. Although both experiments tested college-age students, Experiment 1 was conducted in person, whereas Experiment 2 was conducted online. As the literature on marijuana displayed unhealthy effects of marijuana consumption/reactivity for individuals who were not experiencing health challenges, we expected the FAB to be negatively related to marijuana consumption/reactivity overall in our sample of the general population. As the lone article on FAB and marijuana consumption showed smaller FAB for marijuana consumers than non-marijuana consumers for non-marijuana events, we expected the same finding. Additionally, we expected to find general healthy outcomes in the form of a robust FAB that is positively related to healthy/adaptive variables and negatively related to unhealthy/non-adaptive variables. Verifying the relationship between the FAB and healthy/unhealthy outcomes in the same sample as the investigation of the relationship of the FAB to marijuana consumption will allow for further support for results indicating the adaptive or maladaptive nature of marijuana consumption in a general population.

## 2. Experiment 1

Experiment 1 was conducted in a laboratory, where we asked participants to remember two pleasant and two unpleasant marijuana and non-marijuana events (eight events in total). Participants provided their demographics, estimated their marijuana consumption, rated their traits and emotions, and remembered, described, and rated their events in an order that was balanced with a Latin square. The main dependent variable was the fading of affect for pleasant and unpleasant events. The predictor measures included marijuana consumption, marijuana reactivity, neuroticism, positive and negative affect, depression, anxiety, stress, grit, and sleep disturbance. We expected robust FAB effects that were positively and negatively related to healthy and unhealthy variables, respectively.

### 2.1. Method

#### 2.1.1. Participants

The initial study sample included 328 undergraduates who signed up using the SONA recruitment system from a small, public, liberal arts university in the southeastern United States. The participants ranged in age from 18 to 23 years old, with 1 participant being 41 years old (*M* = 19.52, *SE* = 0.037). Participants were primarily recruited from introductory psychology courses, and they earned course credit for their participation. The sample predominantly contained female (80.6% women), Caucasian (74.5%), Christian (74.6%), and heterosexual (87.8%) individuals. The current study was approved by the university’s Institutional Review Board (IRB# 1349053-2), and participants were treated ethically according to American Psychological Association (APA) guidelines, which included briefing, informed consent, and debriefing (APA, 2017).

#### 2.1.2. Materials and Measures

Materials for the current study included several questionnaires. Specifically, the demographic questionnaire asked participants to describe their gender, race, ethnicity, and religious affiliation. The next questionnaire asked participants about their general marijuana usage, including the hours they spent high, the degree of highness, the number of highness sessions, and the mental competency for each day of the week in a typical week. The questionnaires also included the Big Five Mini-Markers Scale, the Positive and Negative Affect Schedule (PANAS), the Depression, Anxiety, and Stress Survey (DASS), as well as the Grit scale, the Pittsburgh Sleep Quality Index (PSQI), and an event questionnaire. The event questionnaire asked participants to provide event descriptions and then the initial and final affect for each event, the frequency they thought or talked about the event, the cognitive competency for the event, as well as the hours high, the degree of highness, the hours cognitively competent, their highness cognitive competency, and their event cognitive competency for each marijuana event.

**Daily marijuana involvement (consumption and its effects).** Daily marijuana involvement included daily hour high, daily degree of highness, daily number of sessions, and overall cognitive competency. Participants provided the number of hours they spent consuming marijuana on a typical day each day in a typical week, and we calculated the average hours per day. Participants also provided their degree of highness on an average day each day in an average week on a scale ranging from 0 (*completely sober*) to 100 (*completely stoned*), and we calculated the average degree of highness each day. Participants provided their number of marijuana consumption sessions on a typical day each day in a typical week, and we calculated the average number of daily sessions. Participants provided their overall cognitive competency on the day of the experiment on a scale ranging from 0 (*completely incompetent*) to 100 (*completely competent*). The daily marijuana involvement questionnaire was not an existing validated measure, but rather a collection of self-report questions chosen to measure the participant’s perceived level of involvement and usage. The subjectivity of the responses provided should be recognized, as individuals may experience inaccuracies in their memory regarding marijuana usage due to the effects of marijuana usage.

**Neuroticism.** The current study used the entire brief version of the Big Five, called Mini Markers (Saucier, 1994), but we only focused on the 8-item neuroticism scale. The scale asks participants to rate the extent to which they believe adjectives describe them (envious, fretful, jealous, moody, temperamental, touchy, relaxed, unenvious), on a 9-point scale, ranging from 1 (*Extremely Inaccurate*) to 9 (*Extremely Accurate*). The eight items from the neuroticism component of the scale were evaluated, reverse-scored when necessary (items 26 and 36), and used to calculate an average score in the current study, with high scores indicating high neuroticism. Cronbach’s alpha for the neuroticism scale was 0.728.

**Positive and negative affect.** The Positive and Negative Affect Schedule (PANAS) is a 20-item questionnaire that assesses the way participants feel at the time they complete the questionnaire (Watson et al., 1988). The PANAS comprises two mood scales that examine positive and negative affect by instructing participants to rate words, such as “interesting” and “irritable,” respectively, on a 5-point scale, ranging from 1 (*slightly or not at all*) to 5 (*extremely*). Average positive and negative affect scores were calculated, and the Cronbach’s alphas for the positive and negative scales were 0.872 and 0.834, respectively.

**Depression, anxiety, and stress.** The Depression, Anxiety, Stress Scale (DASS-21; Lovibond & Lovibond, 1995) is a 21-item scale assessing depression, anxiety, and stress. Each emotional construct is measured with 7 items. All items used 4-point response scales, ranging from 0 (*did not apply to me at all*) to 3 (*applied to me very much or most of the time*). An example of a depression item is “I found it difficult to work up the initiative to do things.” An average depression score was calculated, and high scores indicated high levels of depression. Anxiety items are scored the same way as depression scores, and an example of an anxiety item is “I felt I was close to panic.” An average anxiety score was calculated, with high scores indicating high levels of anxiety. Stress items are scored the same way as depression and anxiety, and an example of a stress item is “I found it hard to wind down.” An average stress score was calculated, and high scores indicated high levels of stress. Cronbach’s alphas for depression, anxiety, and stress were 0.894, 0.796, and 0.775, respectively.

**Grit.** The grit scale uses 12 items to assess passion, perseverance, and focus in relation to long-term goals (Duckworth & Quinn, 2009). An example of a statement is “Setbacks don’t discourage me. I don’t give up easily.” Each item was rated on a 5-point response scale ranging from 1 (*not like me at all*) to 5 (*very much like me*). Items 1, 3, 5, 7, 9 were reverse scored and the average was calculated, with high scores indicating high levels of grit. Cronbach’s alpha for average grit was 0.810.

**Sleep.** The Pittsburgh Sleep Quality Index (PSQI) is a questionnaire with open-ended questions and Likert-type statements used to measure sleep quality and sleep disturbances. The open-ended questions approached sleep duration with four questions, such as “During the past month, what time have you usually gone to bed at night?” Additionally, the seven closed-ended questions asked for the frequency of sleep disturbances with statements, such as, “During the past month, how often have you had trouble sleeping because you cannot get to sleep within 30 min?” Answers were given on a 4-point Likert-type scale ranging from 1 (*Not during the past month*) to 4 (*Three or more times a week*). Furthermore, participants were given a space to name any disturbance not named by the questionnaire, with a scale providing the frequency with which the event described by the item was experienced. The scores from the items were averaged, and Cronbach’s alpha for the PSQI scale was 0.728.

**Fading affect and rehearsal for events.** After each event was briefly described in four lines or less, it was rated for initial and current affect with each one using a single-item scale ranging from −3 (*very unpleasant*) to +3 (*very pleasant*), including a score of 0 (*neutral*). For initially pleasant events, fading affect was calculated by subtracting the current affect from the original affect. For initially unpleasant events, fading affect was calculated by subtracting the original affect from the current affect. These calculations ensured that all measures indicating fading affect were positive across pleasant and unpleasant events, such that a large fading affect score indicated a large amount of fading, and a small fading affect score indicated little fading. Each event was also rated for the frequency it was thought or talked about with a single-item scale ranging from 0 (*never/infrequently*) to 6 (*very frequently*).

**Marijuana involvement per marijuana event.** Marijuana involvement per marijuana event included the number of hours consuming marijuana for that marijuana event and the degree of highness for that event. The degree of highness scale used a 1-item answer scale ranging from 0 (*completely sober*) to 100 (*completely stoned*).

#### 2.1.3. Procedure

Participants signed up for the study through SONA, and they were provided a timeslot to complete the in-person study. Upon arrival, participants received a consent form. Participants were then informed that their consent was being sought for participation in the research study, their participation was completely voluntary, and they could stop participating at any time without any negative repercussions. Participants were also briefed on the fact that they would complete multiple questionnaires concerning general demographics, their marijuana consumption, personality, mood, psychological distress, sleep quality, and grit. Participants were informed that the study examined the emotions tied to pleasant and unpleasant event memories involving and not involving marijuana consumption (theirs or other individuals), along with rehearsal ratings for these events. Participants were told that they should only provide events that did not cause emotional pain, and, therefore, the experimental procedure should have provided no known risks to them.

Participants were informed that the experiment would demand up to 60 min of their time, and they would receive equitable and reasonable class credit for their participation. Participants were told that the information they provided was confidential and would only be examined by research assistants or the Principal Investigator of the study. Participants were also informed that their data would be encrypted and placed under lock and key. Participants were then given the contact information of the Principal Investigator and the Chair of the IRB. Participants were also given the contact information of the university counseling center to be used in the unlikely case that they experienced emotional discomfort. After receiving all the information in the briefing, participants signed the consent form before engaging in the procedure.

Following the briefing, participants completed a packet of questionnaires, which included general demographics, personality, mood, psychological distress, sleep quality, and grit. Then, participants recalled events that occurred within the last 7 days, including pleasant and unpleasant marijuana and non-marijuana events. Participants were told that marijuana events involved the consumption of marijuana by them and/or someone with whom they were engaging. For each event, participants reported the date that the event occurred (as specifically as possible), and they wrote a brief, four-line event description disclosing as much information as they felt comfortable sharing. Participants then provided an initial/original emotion for the way they felt at the time of the event. Participants were told that unpleasant events should initially be rated using a negative number ranging from −3 (*very unpleasant*) to −1 (*mildly unpleasant*) and pleasant events should be initially rated using a positive number ranging from 1 (*mildly pleasant*) to 3 (*very pleasant*). Participants were asked to rate the way they currently (time of the experiment) felt about the event on a scale ranging from −3 (*very unpleasant*) to +3 (*very pleasant*).

Participants then provided the frequency with which they thought or talked about an event on a rating scale ranging from 0 (*never/infrequently*) to 6 (*always/very frequently*). For marijuana events, participants provided the number of hours they consumed marijuana for that event and their degree of highness for that event. Finally, participants were given a debriefing form, asked to read it in its entirety, and asked if they had any questions. Participants were given credit through SONA following their debriefing.

### 2.2. Analytic Strategy

The analytic strategy in the current study was based on many FAB studies and, particularly, the Gibbons et al. (2013) study in which Timothy Ritchie was the main data analyst. Event, not participant, was the unit of analysis, and fading affect was the dependent variable. Although the 328 participants provided 2624 events, participants did not provide initial or final affect ratings for 614 of these events, and they did not provide the correct event for the requested label in terms of pleasantness or marijuana for another 199 events. A total of 1811 events remained for analysis. We first tested the FAB and whether it was moderated by event type using a 2 (Initial Event Affect) × 2 (Event Type) completely between-groups ANOVA with initial event affect (pleasant or unpleasant) and event type (Marijuana or Non-marijuana) as the independent variables, as done in previous research (e.g., Gibbons et al., 2024). The *F*-values, partial eta^2^ values, and *p*-values were reported for these analyses. In the event of a significant Initial Event Affect × Event Type interaction, *t*-values were planned to be used to understand it.

We then present results from clustered data, including a nominal-level variable to represent each participant and control for clustered data in each model. This data structure enabled us to test for systematic differences in fading affect among four types of events as they related to self-reported continuous measures. The continuous variables included positive and negative PANAS, depression, anxiety, stress, neuroticism (as part of the Mini Markers scale), Grit, and poor sleep quality (PSQI). Continuous variables also included cognitive competency, average daily marijuana consumption in hours, average daily highness, and average daily number of sessions. For marijuana events, continuous variables also included the number of hours high, and the degree of highness. We tested the two-way interactions between initial event affect (pleasant vs. unpleasant) and individual difference variables in predicting fading affect, while controlling for relevant main effects, and participant. To protect for the family-wise error rate when examining the two-way interaction effects of 14 continuous variables, a Bonferroni correction was used, which meant that each of these two-way interactions was tested with an individual alpha level of 0.003571. More importantly, we tested the three-way interactions between initial event affect (pleasant vs. unpleasant), event type (marijuana vs. non-marijuana), and continuous marijuana consumption/reactivity variables while controlling for all two-way interactions, relevant main effects, and participant. To protect for family-wise error rate when examining the three-way interactions involving the five marijuana consumption/reactivity measures, a Bonferroni correction was used, which meant that each of these three-way interactions was tested with an alpha level of 0.01.

To test our moderation hypotheses, we employed Model 1 of the Process macro v5.0 via IBM SPSS (Hayes, 2013, 2022) to examine fading affect, *y*, for pleasant and unpleasant events (initial event affect), *x*, across the continuum of self-reported, continuous individual difference variables, *w*, while controlling for the second level variable of individual through the Process macro’s built in cluster robust SE option. If the FAB occurred, the Johnson–Neyman technique, which breaks down the interactive effect of initial event affect on fading affect (i.e., FAB) across 22 sections of the continuous predictor, *m*, indicated if the FAB was evident for individuals who reported low or high levels of an individual difference variable (Preacher et al., 2006). For any statistically significant interaction analyses produced by the Process macro, we reported the indirect effect, the corresponding standard error, *t*-value, *p*-value, 95% CI lower- and upper-estimates, as well as Δ*R*^2^ (effect size) at each level of the moderators.

We also tested the three-way interactions between initial event affect and two-variable combinations of individual difference variables, while controlling for all two-way interactions, relevant main effects, and the nominal-level variable, participant. Using Model 3 of the Process macro (Hayes, 2013, 2022), we specified the control variable (participant), the main effects, the two-way interactions between initial event affect, *x*, event type, *w*, and a self-reported, continuous individual difference variable, *z.* We examined the interactive effect of initial event affect and event type across the self-reported, continuous individual difference variables using the Johnson–Neyman technique. The goal of these analyses was to determine the points where the FAB was large and small for levels of one self-reported, continuous individual difference variable, across levels of another individual difference variable. However, the results of Johnson–Neyman analyses were not reported as they were deemed unnecessarily cumbersome to the results.

### 2.3. Results

#### 2.3.1. Main Effects and Two-Way Interactions

**Figure 1:**
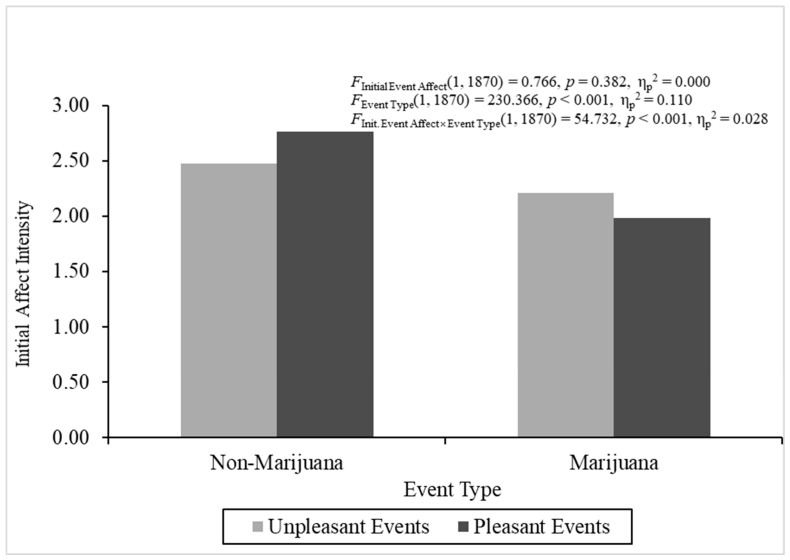
Initial affect intensity across pleasant and unpleasant non-marijuana and marijuana events for Experiment 1.

**The ANOVAs for the discrete predictors.** The ANOVAs produced heterogeneity, but violation of this parametric assumption for conducting ANOVA is not a problem if the sample sizes are relatively equal, defined by a ratio of largest to smallest sample sizes equal to or less than 1.5 (Statistics Solutions, 2023). The sample size ratios calculated for initial event affect, event type, and the interaction were all less than 1.5, and, hence, relatively equal. The overall analysis of variance investigating initial affect intensity was statistically significant, *F*(3, 1870) = 97.978, *p* < 0.001, η_p_^2^ = 0.136. Pleasant events (*M* = 2.431, *SE* = 0.026) did not demonstrate different initial affect intensity than unpleasant events (*M* = 2.362, *SE* = 0.026), *F*(1, 1870) = 0.766, *p* > 0.38, η_p_^2^ = 0.000, which did not support regression-to-the-mean as an explanation for FAB effects. The non-marijuana events (*M* = 2.620, *SE* = 0.020) were initially more intense than the marijuana events (*M* = 2.090, *SE* = 0.030), *F*(1, 1870) = 230.366, *p* < 0.001, η_p_^2^ = 0.110. In addition, the Initial Event Affect × Event Type interaction was statistically significant, *F*(1, 1870) = 54.732, *p* < 0.001, η_p_^2^ = 0.028. Figure 1 depicts initial affect intensity across initial event affect and event type.

**Figure 2:**
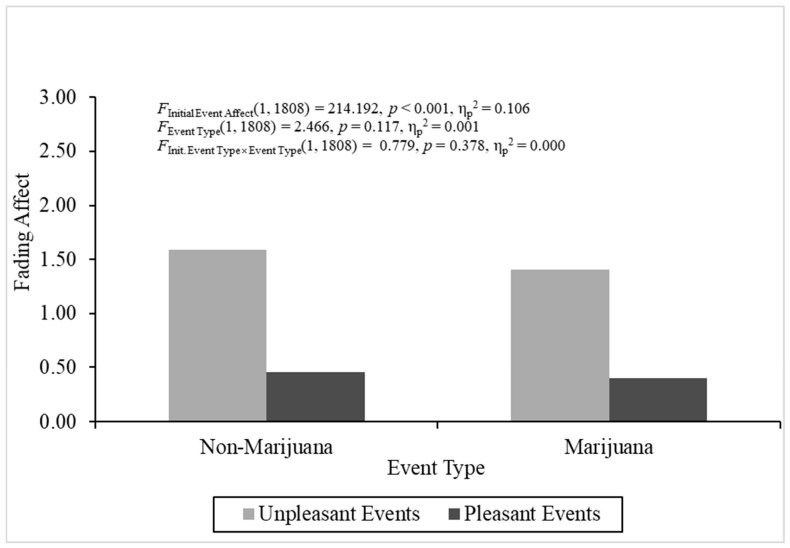
Fading affect across pleasant and unpleasant non-marijuana and marijuana events for Experiment 1.

The overall analysis of variance for fading affect was statistically significant, *F*(3, 1808) = 75.768, *p* < 0.001, η_p_^2^ = 0.112. Importantly, affect faded more for unpleasant events (*M* = 1.517, *SE* = 0.058) than pleasant events (*M* = 0.433, *SE* = 0.043), *F*(1, 1808) = 214.192, *p* < 0.001, η_p_^2^ = 0.106, which supported the hypothesis that a robust FAB effect would be found. No other effects were significant in the ANOVA. Figure 2 depicts the fading affect across initial event affect and event type.

**Process Model 1 analyses for continuous predictors.** We used Process Model 1 (Hayes, 2013, 2022) to examine whether self-reported, continuous individual variables predicted the FAB. As one major goal of the study was to test whether marijuana consumption/reactivity variables were negatively related to the FAB as found by Pillersdorf and Scoboria (2019), we examined each of these a priori predicted relations at an unprotected alpha level of 0.05. However, we evaluated the remaining nine continuous measures as predictors of FAB using a Bonferroni correction to evaluate each effect at an alpha level of 0.0056. The significant continuous marijuana consumption/reactivity predictors of the FAB were positive, and they included cognitive competency and highness for marijuana events. The remaining significant continuous predictors of the FAB were negative and included negative PANAS, depression, anxiety, and sleep disturbance.

For the cognitive competency variable, Process Model 1 (Hayes, 2022) revealed a significant main effect of cognitive competency as well as a significant two-way interaction between cognitive competency and initial event affect (i.e., FAB), B = 0.010 (*SE* = 0.003), *t*(1705) = 2.925, *p* < 0.004, 95% CI [0.003, 0.017], Model Δ*R*^2^ (due to the two-way interaction) = 0.007, overall Model *R*^2^ = 0.121, *p* < 0.01. The FAB increased with cognitive competency primarily because the fading of unpleasant affect increased as cognitive competency increased. For highness of marijuana events, Process Model 1 revealed a significant main effect of marijuana event highness as well as a significant two-way interaction between marijuana event highness and initial event affect, B = 0.011 (*SE* = 0.005), *t*(714) = 2.456, *p* < 0.015, 95% CI [0.004, 0.020], Model Δ*R*^2^ (due to the two-way interaction) = 0.010, overall Model *R*^2^ = 0.094, *p* < 0.001. The FAB increased with marijuana event highness primarily because the fading of pleasant affect decreased as marijuana event highness increased.

When examining negative PANAS as a predictor of the FAB (e.g., initial event affect), Process Model 1 revealed significant main effects for initial event affect and negative PANAS. Moreover, the results from Process Model 1 revealed a significant two-way interaction between negative PANAS and initial event affect, B = −0.670 (*SE* = 0.106), *t*(1802) = −4.531, *p* < 0.001, 95% CI [−0.962, −0.379], Model Δ*R*^2^ (due to the two-way interaction) = 0.019, overall Model *R*^2^ = 0.130, *p* < 0.001. The FAB decreased with negative PANAS because the fading of unpleasant affect increased and the fading of pleasant affect decreased as negative PANAS increased.

For depression, Process Model 1 revealed significant main effects of depression and initial event affect as well as a significant two-way interaction between depression and initial event affect, B = −0.642 (*SE* = 0.144), *t*(1808) = −4.454, *p* < 0.001, 95% CI [−0.926, −0.358], Model Δ*R*^2^ (due to the two-way interaction) = 0.015, overall Model *R*^2^ = 0.127, *p* < 0.001. The FAB decreased with depression primarily because the fading of unpleasant affect decreased as depression increased. For anxiety, Process Model 1 revealed significant main effects of anxiety and initial event affect as well as a significant two-way interaction between anxiety and initial event affect, B = −0.501 (*SE* = 0.158), *t*(1808) = −3.173, *p* = 0.0017, 95% CI [−0.811, −0.190], Model Δ*R*^2^ (due to the two-way interaction) < 0.010, overall Model *R*^2^ = 0.122, *p* < 0.001. The FAB decreased with anxiety primarily because the fading of unpleasant affect decreased as anxiety increased.

For poor sleep as measured by the PSQI, Process Model 1 revealed significant main effects of poor sleep and initial event affect as well as a significant two-way interaction between poor sleep and initial event affect, B = −0.638 (*SE* = 0.223), *t*(1703) = −2.860, *p* = 0.0046, 95% CI [−1.077, −0.199], Model Δ*R*^2^ (due to the two-way interaction) < 0.007, overall Model *R*^2^ = 0.120, *p* < 0.001. The FAB decreased with poor sleep primarily because the fading of unpleasant affect decreased as poor sleep increased.

#### 2.3.2. Process Model 3 for Three-Way Interactions with Continuous Predictors

**Figure 3:**
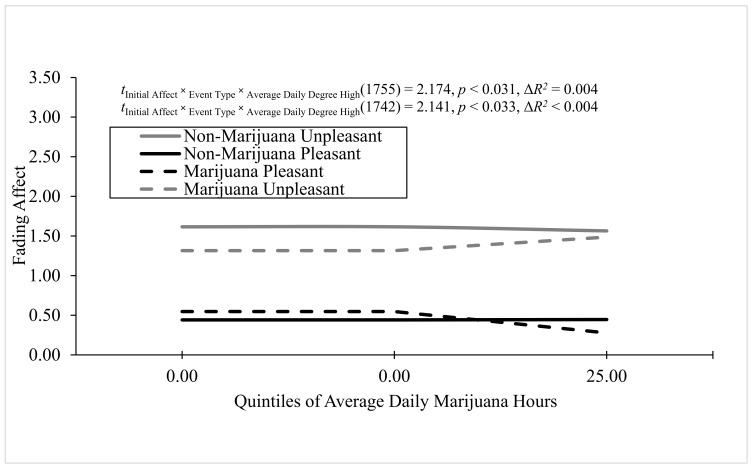
Fading affect for pleasant and unpleasant non-marijuana and marijuana events across quintiles (10th through 90th) of daily marijuana hours for Experiment 1.

The other major goal of the study was to examine the predicted negative relations of the five continuous marijuana consumption/reactivity measures to the FAB across marijuana and non-marijuana events based on the oversight by Pillersdorf and Scoboria (2019). Therefore, we evaluated these three-way interactions involving marijuana consumption/reactivity using an uncorrected alpha level of 0.05, and all other three-way interactions were ignored. We found significant three-way interactions for average daily hours spent consuming marijuana and average daily degree of highness, and the three-way interaction involving average daily sessions spent consuming marijuana approached but did not reach statistical significance. For average daily highness, Process Model 3 revealed a significant main effect of initial event affect as well as significant Event Type × Daily Highness and Initial Event Affect × Event Type two-way interactions. Importantly, the three-way interaction between average daily highness, event type, and initial event affect was significant, B = 0.199 (*SE* = 0.009), *t*(1755) = 2.174, *p* = 0.031, 95% CI [0.002, 0.038], Model Δ*R*^2^ (due to the three-way interaction) < 0.004, overall Model *R*^2^ = 0.119, *p* < 0.001. As seen in Figure 3, the FAB started large and significant for non-marijuana events and did not change with average daily highness, whereas the FAB started significant and smaller for marijuana events and increased significantly as average daily highness increased.

For average daily marijuana hours, Process Model 3 (Hayes, 2013, 2022) revealed significant main effects of average daily marijuana hours and initial event affect. In addition, two of three two-way interactions (initial event affect × daily hours, event type × highness) were significant and, importantly, the three-way interaction between average daily marijuana hours, event type, and initial event affect was significant, B = 0.209 (*SE* = 0.098), *t*(1742) = 2.141, *p* = 0.033, 95% CI [0.017, 0.401], Model Δ*R*^2^ (due to the three-way interaction) = 0.004, overall Model *R*^2^ = 0.118, *p* < 0.001 (i.e., Figure 3). The FAB started large and significant for non-marijuana events and did not change, as average daily marijuana hours increased. In contrast, the FAB started positive and significant, but a little smaller for marijuana events, and increased slightly as average daily marijuana hours increased.

### 2.4. Discussion

We found a robust overall fading affect bias (FAB) that did not differ across marijuana and non-marijuana events, as exhibited via the absence of a significant Initial Event Affect by Event Type interaction (*F* < 1). Furthermore, overall cognitive competency and marijuana event highness positively predicted the FAB, whereas negative PANAS, depression, anxiety, stress, sleep disturbance, and neuroticism all negatively predicted the FAB. Except for the relation involving the marijuana event highness measure, these findings replicated the outcomes from a variety of FAB studies (e.g., Ritchie et al., 2009; Skowronski et al., 2004; Walker et al., 2014). These results also demonstrated general healthy coping for the FAB.

Extending the marijuana literature showing that marijuana consumption/reactivity primarily acted as unhealthy/non-adaptive variables for healthy individuals, Pillersdorf and Scoboria (2019) found that the FAB was larger for non-marijuana users than for marijuana consumers. However, the results of Experiment 1 showed that (1) marijuana consumption did not significantly predict the FAB, whereas (2) marijuana reactivity in the form of highness for marijuana events positively predicted the FAB. Neither of these results replicated the results for healthy marijuana consumers in the literature, and they did not support the results of Pillersdorf and Scoboria. One explanation for these different findings may pertain to the fact that Experiment 1 examined marijuana and non-marijuana events, whereas Pillersdorf and Scoboria only evaluated non-marijuana events.

The event type explanation is partially supported by the negative, albeit non-significant, relations between the FAB and the three marijuana-consumption measures (average daily marijuana hours and average daily highness) for non-marijuana events as part of the three-way interactions in Experiment 1. These non-significant negative relations for non-marijuana events mirrored the significant negative relation between marijuana consumption and the FAB for the non-marijuana events in the Pillersdorf and Scoboria (2019) study. Experiment 2 was designed to replicate the procedure in Experiment 1 using an online data collection format to determine whether the results in Experiment 1 were (1) an aberration or (2) a direct product of the methodology.

In addition to the various two-way interactions, two three-way interactions involving one marijuana consumption measure (average daily marijuana hours) and one marijuana reactivity measure (average daily highness) were significant, as they differentially predicted the FAB across marijuana and non-marijuana events. Specifically, marijuana consumption positively and significantly predicted the FAB for marijuana events, and negatively and non-significantly predicted the FAB for non-marijuana events. As the negative relations between the FAB and marijuana consumption/reactivity for non-marijuana events were not significant, the results of Experiment 1 did not replicate the results of Pillersdorf and Scoboria (2019).

## 3. Experiment 2

Experiment 2 was designed to replicate the procedure in Experiment 1, but it was conducted online rather than in person. Again, participants were asked to provide demographics, ratings of their traits, estimations of their marijuana consumption, and they were asked to remember, describe, and rate two pleasant and two unpleasant events that involved or did not involve marijuana, totaling eight events, in an order that was balanced with a Latin square. The ratings were the same in Experiment 2 as in Experiment 1. We expected general healthy coping, such that healthy variables were expected to positively predict the FAB and unhealthy variables were expected to negatively predict the FAB. Although the Pillersdorf and Scoboria (2019) finding suggested that we would find a negative relation between marijuana consumption/effects and the FAB, the findings in Experiment 1 suggested that we would find positive relations between the variables. Based on the three-way interactions in Experiment 1, we expected the marijuana consumption/reactivity to (1) positively predict the FAB for marijuana events and (2) negatively predict the FAB for non-marijuana events.

### 3.1. Methods

#### 3.1.1. Participants

The initial study sample included 232 students who signed up using the Qualtrix survey-creation system on Amazon MTurk, which is an online data collection mechanism. Participants ranged in age from 18 to 23 years old (*M* = 21.310, *SE* = 0.039). The age range for the experiment was chosen to match the ages of participants in Experiment 1 to maximize similarity of the samples, The sample primarily comprised female (67.7% women), Caucasian (62.5.5% Caucasian, 20.7% Asian, 9.9% Hispanic, 5.2% African American), Christian (71.1% Christian, 11.2% Hindu, 4.7% Atheist, 3.9% Agnostic, 3% Islam), heterosexual (69.0% Heterosexual, 25.0% Bisexual, 3.9% Homosexual) individuals. The current study was approved by the University Institutional Review Board (IRB# 1576077-2), and participants were treated ethically according to American Psychological Association (APA) guidelines, which included briefing, informed consent, and debriefing (APA, 2017).

#### 3.1.2. Materials, Measures, Procedure, and Analytic Strategy

The materials and measures used in Experiment 1 were also used in Experiment 2; the descriptions for these measures can be found in the Materials and Measures section of Experiment 1. The scaled measures included average daily hours of marijuana consumption (α = 0.908), average daily marijuana sessions (α = 0.934), average daily degree of highness (α = 0.961), neuroticism from the Big Five Mini-Markers Scale (α = 0.811), positive PANAS (α = 0.842), negative PANAS (α = 0.872), as well as depression (α = 0.833), anxiety (α = 0.826), stress (α = 0.799), Grit (α = 0.842), and poor sleep as measured by the Pittsburgh Sleep Quality Index (α = 0.796). Scaled measures also included 1-item scales, which pertained to the degree of highness for marijuana events, as well as cognitive competency, initial and current affect, and rehearsal frequency for each event. Non-scaled measures included hours spent consuming marijuana and sessions consuming marijuana for each marijuana event. The procedure in Experiment 2 was the same as the one used in Experiment 1, except participants signed up for the experiment via Amazon MTurk, they clicked a button to provide signed consent, and they completed the entire experiment in an online format. The analytic strategy in Experiment 2 was the same as the one used in Experiment 1.

Two research assistants independently rated marijuana and non-marijuana events as 1 (*description clearly matched label*), 2 (*description likely matches label*), 3 (*description might match label*), 4 (*undescribed*), and 5 (*description matches opposite event label*). The kappa for interrater reliability was 0.778 (*SE* = 0.011), *p* < 0.001. The percent agreement for the two research assistants was 83.351%. From the 1856 events, we removed 128 events that were rated a 5 by either rater, as these events clearly did not match their event label.

### 3.2. Results

#### 3.2.1. Main Effect and Two-Way Interactions: Evidence of General Healthy Coping for FAB

**Figure 4:**
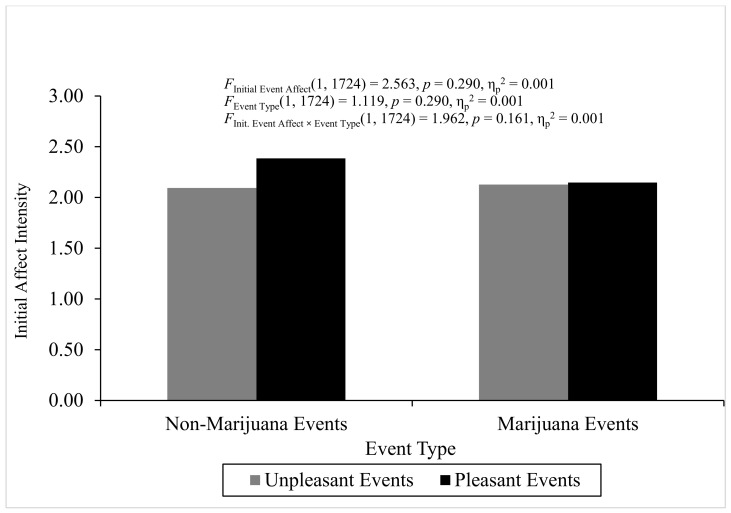
Initial affect intensity for pleasant and unpleasant non-marijuana and marijuana events for Experiment 2.

**Figure 5:**
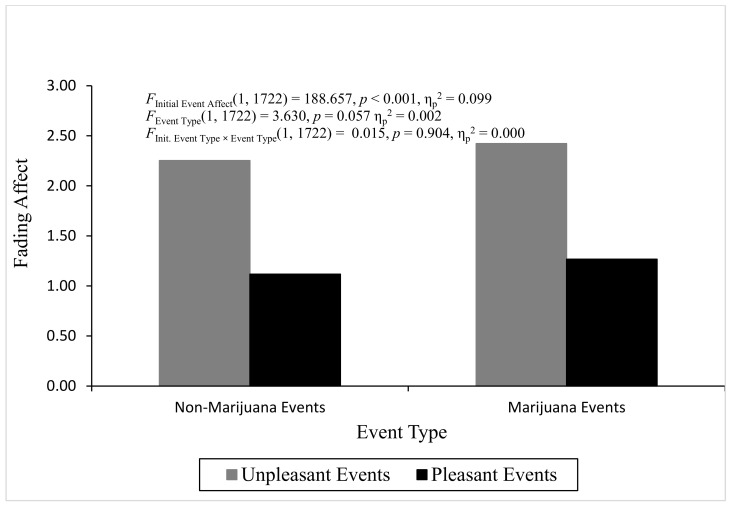
Fading affect for pleasant and unpleasant non-marijuana and marijuana events for Experiment 2.

**ANOVA analyses for discrete two-way interactions.** The ANOVA for initial affect intensity did not produce heterogeneity. The overall analysis of variance for initial affect intensity was not statistically significant, *F*(3, 1724) = 1.804, *p* = 0.144, η^2^_partial_ = 0.003, and the two main effects and interaction were not statistically significant (*p* > 0.05), which means that regression-to-the-mean could not explain any significant FAB effects. Figure 4 displays initial event affect across event type and initial event affect. The ANOVA for fading affect did produce significant heterogeneity, but violation of this parametric assumption for conducting ANOVA is not a problem if the sample sizes are relatively equal, defined by a ratio of largest to smallest sample sizes equal to or less than 1.5 (Statistics Solutions, 2023). The sample size ratios calculated for initial event affect, event type, and the interaction were all less than 1.5, and, hence, relatively equal. The overall ANOVA for fading affect was statistically significant, *F*(3, 1722) = 64.128, *p* < 0.001, η^2^_partial_ = 0.100. Only the main effect of initial event affect was statistically significant, *F*(1, 1722) = 188.657, *p* < 0.001, η^2^_partial_ = 0.099, with unpleasant events showing greater fading affect (*M* = 2.341, *SE* = 0.064) than pleasant events (*M* = 1.197, *SE* = 0.053), which demonstrated a fading affect bias (FAB). Figure 5 depicts fading affect across event type and initial event affect.

**Figure 6:**
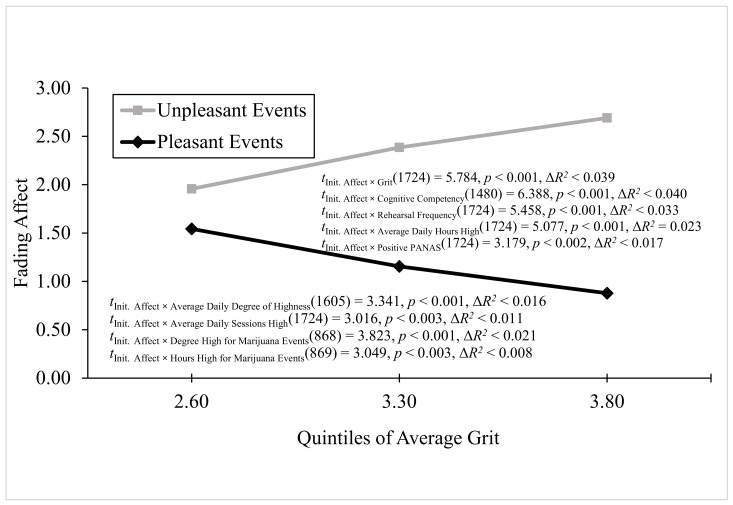
Fading affect for pleasant and unpleasant events across quintiles (10th through 90th) of grit for Experiment 2.

**Process Model for continuous two-way interactions.** We used Process Model 1 (Hayes, 2013, 2022) to examine whether self-reported, continuous individual variables predicted the FAB. As we were replicating the results in Experiment 1 and expecting to find that the marijuana consumption/reactivity variables positively correlated with the FAB, we tested each of those relations at an unprotected alpha level of 0.05. However, all the continuous two-way interactions, except the one involving negative PANAS, were significant using a Bonferroni correction of 0.003571 for the 14 relations. The significant positive predictors of the FAB included grit, cognitive competency, rehearsal frequency, average daily hours high, positive PANAS, average daily degree of highness, average daily sessions high, degree of marijuana event highness, and hours high for marijuana events. For the grit variable, Process Model 1 revealed significant main effects of grit and initial event affect (i.e., FAB) as well as a significant two-way interaction of grit and initial event affect, B = 1.165 (*SE* = 0.202), *t*(1724) = 5.784, *p* < 0.001, 95% CI [0.768, 1.562], Model Δ*R*^2^ (due to the two-way interaction) = 0.039, overall Model *R*^2^ = 0.138, *p* < 0.001. Figure 6 shows that the FAB increased with grit because the fading of pleasant events decreased, and the fading of unpleasant events increased as grit increased.

For the cognitive competency variable, Process Model 1 revealed significant main effects of cognitive competency and initial event affect as well as a significant two-way interaction of cognitive competency and initial event affect, B = 0.031 (*SE* < 0.005), *t*(1480) = 6.388, *p* < 0.001, 95% CI [0.022, 0.041], Model Δ*R*^2^ (due to the two-way interaction) = 0.040, overall Model *R*^2^ = 0.135, *p* < 0.001 (i.e., Figure 6). The FAB increased with cognitive competency because the fading of pleasant events decreased, and the fading of unpleasant events increased, but mostly because the fading of pleasant affect decreased as cognitive competency increased.

For the rehearsal frequency variable, Process Model 1 revealed significant main effects of rehearsal frequency and initial event affect as well as a significant two-way interaction of rehearsal frequency by initial event affect interaction, B = 0.385 (*SE* = 0.070), *t*(1724) = 5.458, *p* < 0.001, 95% CI [0.246, 0.523], Model Δ*R*^2^ (due to the two-way interaction) < 0.033, overall Model *R*^2^ = 0.138, *p* < 0.001 (i.e., Figure 6). The FAB increased with rehearsal frequency because the fading of pleasant events decreased with rehearsal frequency, but mostly because the fading of unpleasant events increased with rehearsal frequency.

For average daily hours high, Process Model 1 revealed significant main effects of average daily hours high and initial event affect, as well as a significant two-way interaction between average daily hours high and the initial event affect, B = 0.127 (*SE* < 0.025), *t*(1724) = 5.077, *p* < 0.001, 95% CI [0.078, 0.176], Model Δ*R*^2^ (due to the two-way interaction) = 0.023, overall Model *R*^2^ = 0.125, *p* < 0.001 (i.e., Figure 6). The FAB increased with average daily hours high because the fading of pleasant events decreased, but mostly because the fading of unpleasant events increased as average daily hours high increased. For the positive PANAS variable, Process Model 1 revealed significant main effects of positive PANAS and initial event affect, as well as a significant two-way interaction between positive PANAS and initial event affect, B = 0.580 (*SE* = 0.182), *t*(1724) = 3.179, *p* < 0.002, 95% CI [0.220, 0.939], Model Δ*R*^2^ (due to the two-way interaction) < 0.017, overall Model *R*^2^ < 0.116, *p* < 0.001 (i.e., Figure 6). The FAB increased with positive PANAS because the fading of pleasant events decreased, and the fading of unpleasant events increased as positive PANAS increased.

For average daily degree of highness, Process Model 1 revealed a significant main effect of average daily degree of highness and a significant two-way interaction between average daily degree of highness and initial event affect, B = 0.017 (*SE* = 0.005), *t*(1724) = 3.341, *p* = 0.001, 95% CI [0.007, 0.027], Model Δ*R*^2^ (due to the two-way interaction) = 0.016, overall Model *R*^2^ < 0.126, *p* < 0.001 (i.e., Figure 6). The FAB increased with average daily degree of highness only because the fading of unpleasant affect increased as average daily degree of highness increased. For the average daily high sessions variable, Process Model 1 revealed significant main effects of average daily high sessions and initial event affect as well as a significant two-way interaction between average daily high sessions and initial event affect, B < 0.196 (*SE* = 0.065), *t*(1724) = 3.016, *p* < 0.003, 95% CI [0.068, 0.323], Model Δ*R*^2^ (due to the two-way interaction) < 0.011, overall Model *R*^2^ < 0.121, *p* < 0.001 (i.e., Figure 6). The FAB increased with average daily high sessions only because the fading of unpleasant events increased as average daily high sessions increased.

For the degree of highness for marijuana events variable, Process Model 1 revealed a significant main effect of the degree of highness for marijuana events as well as a significant two-way interaction between the degree of highness for marijuana events and initial event affect, B < 0.022 (*SE* < 0.006), *t*(868) = 3.823, *p* < 0.001, 95% CI [0.011, 0.033], Model Δ*R*^2^ (due to the two-way interaction) < 0.021, overall Model *R*^2^ < 0.123, *p* < 0.001 (i.e., Figure 6). The FAB increased with the degree of highness for marijuana events because the fading of pleasant events decreased, and the fading of unpleasant events increased as the degree of highness for marijuana events increased.

For hours high for marijuana events, Process Model 1 revealed significant main effects of hours high for marijuana events and initial event affect as well as a significant two-way interaction between hours high for marijuana events and initial event affect, B = 0.054 (*SE* = 0.018), *t*(869) = 3.049, *p* < 0.003, 95% CI [0.016, 0.091], Model Δ*R*^2^ (due to the two-way interaction) = 0.008, overall Model *R*^2^ < 0.111, *p* < 0.001 (i.e., Figure 6). The FAB increased with hours high for marijuana events because the fading of pleasant events decreased slightly, and the fading of unpleasant events increased slightly as hours high for marijuana events increased.

**Figure 7:**
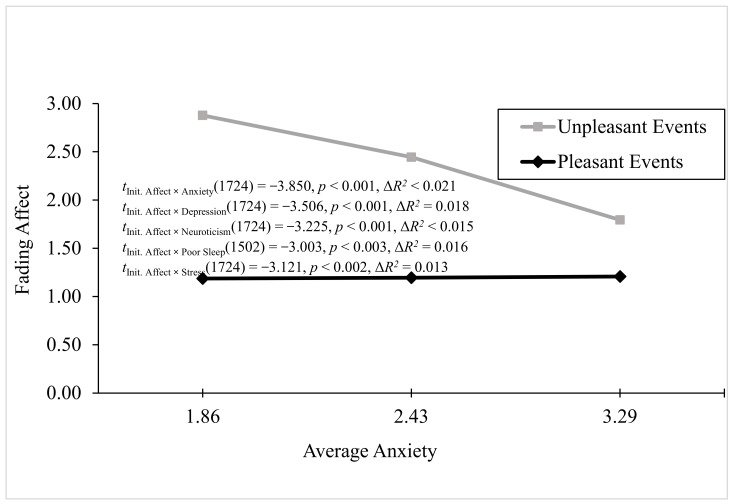
Fading affect for pleasant and unpleasant events across quintiles (10th through 90th) of anxiety for Experiment 2.

The significant negative predictors of the FAB included anxiety, depression, neuroticism, poor sleep, stress, and negative PANAS. For the anxiety variable, Process Model 1 revealed significant main effects of anxiety and initial event affect as well as a significant two-way interaction between anxiety and initial event affect, B = 0.773 (*SE* < 0.201), *t*(1724) = −3.850, *p* < 0.001, 95% CI [−1.169, −0.378], Model Δ*R*^2^ (due to the two-way interaction) < 0.021, overall Model *R*^2^ = 0.139, *p* < 0.001. Figure 7 showed that the FAB decreased with anxiety only because the fading of unpleasant events decreased as anxiety increased. For the depression variable, Process Model 1 revealed significant main effects of depression and initial event affect as well as a significant two-way interaction between depression and initial event affect, B = −0.697 (*SE* = 0.199), *t*(1724) = −3.506, *p* < 0.001, 95% CI [−1.089, −0.306], Model Δ*R*^2^ (due to the two-way interaction) = 0.018, overall Model *R*^2^ < 0.136, *p* < 0.001 (i.e., Figure 7). The FAB decreased with depression only because the fading of unpleasant events decreased as depression increased.

For neuroticism, Process Model 1 revealed significant main effects of neuroticism and initial event affect as well as a significant two-way interaction between neuroticism and initial event affect, B = −0.315 (*SE* = 0.098), *t*(1724) = −3.22, *p* < 0.001, 95% CI [−0.507, −0.122], Model Δ*R*^2^ (due to the two-way interaction) = 0.015, overall Model *R*^2^ = 0.114, *p* < 0.001 (i.e., Figure 7). The FAB decreased with neuroticism because the fading of pleasant affect increased, and the fading of unpleasant events decreased as neuroticism increased. For poor sleep quality, Process Model 1 revealed significant main effects of poor sleep quality and initial event affect as well as a significant two-way interaction between poor sleep quality and initial event affect, B < −0.869 (*SE* = 0.289), *t*(1502) = −3.00, *p* = 0.003, 95% CI [−1.440, −0.299], Model Δ*R*^2^ (due to the two-way interaction) = 0.016, overall Model *R*^2^ < 0.125, *p* < 0.001 (i.e., Figure 7). The FAB decreased with poor sleep quality only because the fading of unpleasant events decreased as poor sleep quality increased.

For the stress variable, Process Model 1 revealed significant main effects of stress and initial event affect as well as a significant two-way interaction between stress and initial event affect, B = −0.646 (*SE* < 0.207), *t*(1724) = −3.121, *p* = 0.002, 95% CI [−1.054, −0.238], Model Δ*R*^2^ (due to the two-way interaction) = 0.013, overall Model *R*^2^ = 0.125, *p* < 0.001 (i.e., Figure 7). The FAB decreased with stress only because the fading of unpleasant affect decreased as stress increased.

#### 3.2.2. Continuous Three-Way Interaction

**Figure 8:**
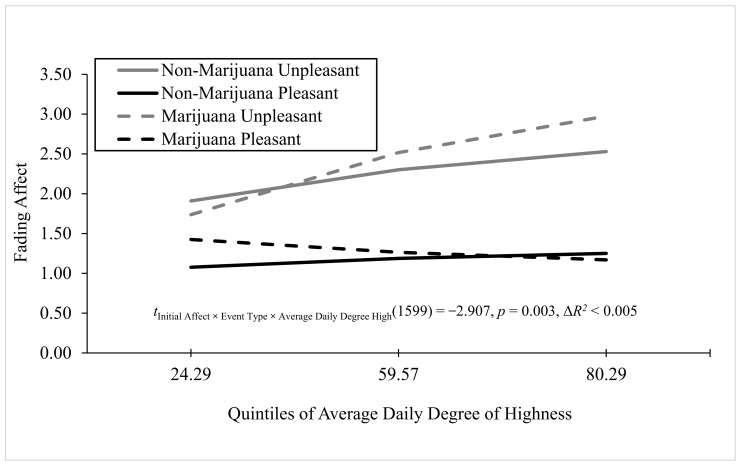
Fading affect for pleasant and unpleasant events across quintiles (10th through 90th) of average daily degree high for Experiment 2.

As we were replicating the two three-way interaction findings from Experiment 1, we evaluated each of them at an unprotected alpha level of 0.05. We found one significant three-way interaction with initial event affect, event type, and a continuous variable measuring marijuana reactivity in the form of average daily degree of highness. For average daily degree of highness, Process Model 3 (Hayes, 2013, 2022) revealed that all the main effects and two-way interactions were significant, as was the three-way interaction between average daily degree of highness, event type, and initial event affect (i.e., FAB), B < −0.019 (*SE* = 0.006), *t*(1599) = −2.907, *p* = 0.004, 95% CI [−0.031, −0.006], Model Δ*R*^2^ (due to the three-way interaction) < 0.005, overall Model *R*^2^ < 0.132, *p* < 0.001. As shown in Figure 8, the FAB started large and significant for non-marijuana events and increased steadily, but not significantly, as average daily degree of highness increased, whereas the FAB started small, negative, non-significant, and reversed for marijuana events and increased strongly and significantly as average daily degree of highness increased.

### 3.3. Discussion

In an online procedure replicating the one used in Experiment 1, Experiment 2 exhibited a robust fading affect bias (FAB) that did not differ across event types, demonstrating general healthy coping and replicating the findings in Experiment 1. Similarly, the continuous measures in Experiment 2 consistently showed general healthy coping, as they significantly predicted the FAB at a Bonferroni-corrected alpha level of 0.003571. Specifically, the FAB was positively and significantly predicted by grit, cognitive competency, rehearsal ratings, average daily hours high, positive PANAS, average degree of highness, average daily high sessions, degree of highness for marijuana events, and hours high for marijuana events, even after the Bonferroni correction was applied to the alpha level. In contrast, the FAB was negatively and significantly predicted by anxiety, depression, neuroticism, poor sleep quality, and stress. These results suggest that the FAB is a form of general healthy coping, which replicates the findings in Experiment 1 and aligns with the general literature consensus on the FAB being a form of healthy coping.

Importantly, every marijuana consumption/reactivity measure positively and significantly predicted the FAB in Experiment 2, which suggested that marijuana consumption/reactivity may be considered adaptive because they were positively associated with healthy emotional coping in the form of the FAB. One reason that marijuana consumption/reactivity could have been perceived as healthy by the online participants in Experiment 2 is that these individuals consumed marijuana more often and were high more frequently and, consequently, enjoyed those experiences more than the individuals in Experiment 1. Except for participants’ degree of highness for marijuana events, post hoc examinations of the marijuana/consumption effects from both experiments supported this explanation. Specifically, the other four measures of marijuana consumption/reactivity (hours high for marijuana events, average daily hours high, average daily degree of highness, and average daily marijuana sessions) were significantly higher in Experiment 2 than in Experiment 1. These results also supported the premise that the online procedure may have allowed participants to openly share their event descriptions as well as their thoughts and feelings about those events to a greater degree than the in-person procedure, which increased the marijuana consumption/effects ratings. Online marijuana consumers may have preferred the privacy of their room or a setting of their choosing to share their events, feelings, and behaviors, unlike in-person marijuana consumers, who may have worried about sharing their illegal activities in a laboratory setting.

More continuous predictors of the FAB were significant in Experiment 2 than in Experiment 1, and with fewer participants, which suggests that the online procedure produced data with less error variance than the in-person procedure. The online procedure in Experiment 2 may have allowed participants to feel secure that their information would remain confidential, increasing the validity and, consequently, the consistency of their responses. A post hoc comparison of the variance statistics (e.g., standard deviations) across the two experiments did not support this possibility, as the results were mixed. Alternatively, participants engaging in the online procedure of Experiment 2 may have been motivated to begin and execute it with effort due to its expedited access. In contrast to the in-person procedure, the online procedure could be accessed at any time and place with internet access, and it did not necessitate travel to the laboratory. Furthermore, the percentage of participants who reported being non-users of marijuana was 62% in the in-person sample while only being 6% in the online sample. This difference in the percentage of marijuana users may have contributed to the number of significant effects in the online sample, as there was a greater frequency and continuity of marijuana usage than in the in-person sample of Experiment 1. Regardless of the explanation, the two-way interaction findings highlight the effectiveness of online studies in FAB research and promote their use in future FAB studies.

In addition to the many two-way interactions, we found one significant three-way interaction in Experiment 2 that involved marijuana reactivity. Specifically, average daily degree of highness was positively related to the FAB for marijuana events and non-marijuana events, but the relation was not significant for non-marijuana events. These results partially replicated the marijuana consumption/reactivity results from Experiment 1, which demonstrated the same significant positive relation for marijuana events. However, the pattern of findings for the non-marijuana events did not replicate across the two experiments in the current study. The in-person and online procedures used in the two experiments may explain the different results. As stated previously, participants online may have felt free to retrieve and describe events from wider contexts than participants in person, which could have influenced their ratings of event affect as well as their ratings of marijuana consumption/effects. The difference in the percentage of the participants that were active marijuana users across the two samples may have also led to the difference in significant relations. The previously mentioned post hoc comparison of most marijuana consumption measures across the two experiments/procedures supported this explanation. This explanation is also supported by post hoc analyses demonstrating higher introversion in Experiment 2 than Experiment 1, a negative relation between introversion and both degree of high for marijuana events and average daily degree of highness in Experiment 1, and a positive relation between introversion and average daily degree of highness in Experiment 2.

The two-way interactions and the three-way interaction involving marijuana reactivity suggest that consuming marijuana and getting high may help people regulate their emotions by quickly losing their unpleasant feelings for marijuana events while holding onto their pleasant feelings for marijuana events, with the likely consequence being further marijuana engagement. Alternatively, emotional regulation in the form of faded unpleasantness and maintained pleasantness could prompt individuals to consume marijuana or a third variable may account for both variables. Future research can test the premise of a causal relation between the FAB and marijuana engagement by tracking marijuana novices who have just begun to consume marijuana across time. These individuals should describe their marijuana and non-marijuana events, as well as their marijuana consumption/effects across time (e.g., 6 to 12 weeks). This quasi, cross-lagged panel design could help determine if early FAB leads to later marijuana consumption/effects and events or early marijuana consumption/effects and events lead to later FAB. Similar studies could be run in the context of any drug or behavior that could be considered habitual or addictive, such as alcohol, nicotine, caffeine, videogame play, social media, television shows, movies, and gambling. The problem with such a study is the pressure that participants may feel to continue the newly introduced activity. Therefore, participants in such an experiment should be continually reminded by the experimenters that they can quit engaging in the addictive behavior throughout the entire experimental timeline.

## 4. General Discussion

We found robust overall fading affect bias (FAB) effects in Experiments 1 and 2 that did not differ across marijuana and non-marijuana events. In addition, the FAB in both experiments was positively and negatively related to healthy/adaptive and non-healthy/maladaptive measures, respectively. These findings replicated and extended past work showing similar results (Ritchie et al., 2009; Skowronski et al., 2004; Walker et al., 2014).

The positive relations between marijuana consumption/reaction measures and the FAB in both experiments ran counter to the expectations based on the FAB being higher for non-marijuana than marijuana consumers in the Pillersdorf and Scoboria (2019) study. One possible explanation for these different findings is that the FAB was not compared across marijuana consumers and non-marijuana consumers in both experiments in the current study. Therefore, we compared fading affect for pleasant and unpleasant events across participants who did and did not consume marijuana or did and did not get high for every marijuana consumption/reactivity measure. For every measure, we found that pleasant affect faded less or unpleasant affect faded more for marijuana consumers reporting being high than marijuana non-consumers not reporting being high. These results demonstrated a positive relation between marijuana consumption/reactivity and the FAB, and they are in line with the two-way interactions displaying that relation in the current study, not the results from Pillersdorf and Scoboria (2019) showing a larger FAB for marijuana non-consumers than marijuana consumers. Although the samples in the two studies differed in many ways, including the fact that 38% of participants reported being marijuana users in Experiment 1, whereas 94% of participants reported being marijuana users in Experiment 2, the results were very similar across the two experiments. Therefore, the difference in the findings of the current study and the Pillersdorf and Scoboria study cannot be attributed to differences in the samples in the current study. In fact, the difference in usage rates between samples used in the two experiments in the current study extends the generalizability of the common findings.

Even though two three-way interactions involving marijuana consumption/reactivity and event type significantly predicted the FAB in Experiment 1, only the three-way interaction involving marijuana reactivity (average daily degree of highness) was significant in Experiment 2. The strong two-way interactions in Experiment 2 could have driven the marijuana consumption/effects to positively predict the FAB in consistent ways across marijuana and non-marijuana events that limited their influence on these strong relations. A more plausible explanation for the lone three-way FAB interaction in Experiment 2 compared to the two three-way interactions in Experiment 1 is the fact that an individual’s cannabis consumption/effects depend largely on their dependency. Prior research has shown that individuals who meet the full diagnostic criteria for cannabis dependency tend to experience many negative effects, including particularly poor cognitive functioning and mental health (Braidwood et al., 2018; Looby & Earleywine, 2007). In contrast to non-dependent users, who do not show the same mental health issues (Braidwood et al., 2018; Degenhardt et al., 2002; van der Pol et al., 2013) and may experience FAB as a healthy or adaptive coping mechanism, dependent users may experience FAB as an avoidant coping mechanism that could be connected to poor mental health. This dependency explanation is in line with the fact that more participants consumed marijuana regularly in Experiment 2 (94%) than in Experiment 1 (38%). As the current study did not test for this potentially influential factor, future research testing FAB effects in the context of addictive behaviors should measure it and statistically control for it.

A limitation related to dependency is the fact that the current study did not differentiate between participants’ reported use of marijuana as recreational and/or medicinal. Although recreational users may show higher FAB than medicinal users because they choose to consume marijuana to a greater degree than medicinal users who may be guided by their condition to consume marijuana and reap its beneficial effects. However, the literature in the introduction suggested that marijuana consumption has produced adaptive effects in individuals struggling to maintain their health, whereas marijuana consumption seems to lead to maladaptive outcomes for healthy users. Therefore, future research should answer this research question.

The instability of the three-way interactions, paired with the small effect sizes (e.g., Model Δ*R*^2^ 0.004 to 0.005) for the three-way interactions should be considered as a potential weakness of the current study’s findings and a point for which further research should be conducted. While small effect sizes as the ones found in the three-way interactions presented here may be valuable in an epidemiological sense, considering the wide-spread usage of marijuana, the scale of the current study does not meet the general standards to support epidemiologically valid effects. Thus, despite statistical significance, the effect sizes of the three-way interactions found may have limited practical implications. To enhance these effect sizes, future research should consider manipulations, such as the presentation of research on the adaptive or maladaptive effects of marijuana consumption, that will enhance FAB effects. In addition, these manipulations could be examined in the context of the laboratory or for different groups of participants who engage in a longitudinal diary study of their marijuana consumption/reactivity and affective responses to events they experience for one to four weeks. A related limitation in the current study is the fact that it used a retrospective autobiographical memory procedure, which can be problematic as the memories from these procedures can be partially or entirely reconstructed. Although diary memory studies are an excellent alternative to retrospective memory procedures in terms of accuracy, Ritchie et al. (2009) found in a combined diary and retrospective memory procedure that participants estimated the initial intensity of pleasant events accurately and they underestimated the initial intensity of unpleasant events. In other words, this study showed that retrospective memory procedures underestimated the actual FAB, which is preferable to an overestimation bias.

One major limitation in the current study is that we did not ask participants to provide their marijuana reactivity, which includes the degree of highness and hours high, for non-marijuana events. Participants who spent a majority of their time high could have been, and likely were, under the influence of marijuana for their non-marijuana events. Therefore, the variables measuring marijuana reactivity for both event types may have been able to show strong positive relations to the FAB for both marijuana and non-marijuana events, but only for individuals reporting high levels of marijuana involvement. Such an effect would be noted in the breakdown of a significant interaction of initial event affect, daily marijuana involvement, and hours/degree high across both events. Future research should correct for this limitation by measuring marijuana reactivity and analyzing its effects on the FAB within the context of addictive behaviors for both the addictive behavior event (e.g., marijuana) and the non-addictive behavior event (e.g., non-marijuana).

Another limitation is that participants were asked to subjectively assess the way they felt after consuming marijuana. Unfortunately, individuals do not always make these judgments consistently and accurately. For example, King et al. (2021) gave heavy and very heavy intravenous alcohol doses to a group that had been drinking heavily for a prolonged period before the experiment and then tested their physiological and subjective reactions. Although the participants’ physiological reactions were different across the two alcohol doses, their subjective reactions did not differ across them. These results demonstrate that subjective assessments do not always accurately reflect actual drug doses the same way as objective measures, such as physiological reactions. Future studies could employ physiological measures to enhance the accuracy of their measures and the validity of their conclusions. However, physiological measures are often difficult and expensive to administer, and subjective experiences are best captured with subjective measures. Therefore, researchers have to weigh the costs and benefits of using physiological and subjective measures. As an alternative to both these measures, future research could ask participants to engage in challenging tasks (e.g., puzzles) when they are or are not high, and then measure their performance, reaction time, and subjective assessments of their experiences. To avoid the legal and ethical considerations of marijuana manipulations in the laboratory, these studies could be conducted online with participants logging in and providing information at various times when they are high.

Beyond physiological measurement, future research would also benefit from the use of validated and supported measures for marijuana consumption and for cognitive ability. The results presented for the current study are heavily limited by the lack of psychometrically supported measurements of marijuana usage, as well as the study design requiring repeated usage of these unvalidated measures. The lack of a standardized variable decreases the generalizability of the current study and thus leads to a need for further research supporting the findings with existing measures. Additionally, the use of single items to measure these variables may lead to overgeneralization of experiences, thus causing an underspecification of the actual effects both in the data on a personal highness reporting level and in the analyses on a statistical level. Such concerns over single-item measurements are not shared with the affect rating questions, as those items have been used as a standard across FAB literature and have been repeatedly supported as demonstrating construct validity. Although research should always be concerned with and test for regression to the mean explanations for any effects, the FAB has been free from these effects in past research and no evidence for such effects was found in the current study.

In summary, two experiments found general healthy outcomes in the form of significant FAB effects that did not differ across event type, as well as positive and negative relations of the FAB to adaptive and non-adaptive variables, respectively. These two experiments also demonstrated positive relations between marijuana consumption/reactivity and the FAB, as well as significant three-way interactions in which marijuana consumption/reactivity was positively associated with the FAB for marijuana events. These results are important because they contradict the findings of Pillersdorf and Scoboria (2019), who found that the FAB for non-marijuana events was smaller for marijuana consumers than non-marijuana consumers. Limitations included marijuana consumption rate differences between samples, potential differences in marijuana dependency, not assessing recreational vs. medicinal marijuana use, small effect sizes, retrospective procedures, subjective measures, and single-item measures for marijuana consumption/reactivity. Future research should correct these limitations to enhance the internal validity of FAB findings in the context of marijuana and entice other researchers to examine the FAB in similar contexts. In conclusion, the current study found robust FAB, which did not differ across marijuana and non-marijuana events, but its positive relations to marijuana consumption and reactivity were significant for marijuana events but not for non-marijuana events, and these results differed from the seminal FAB study in the context of marijuana.

## Data Availability

The data are available from the first author upon reasonable request.
